# Navigating Personalised Support in Universal Credit: Local Support, Care Responsibilities, and Claimant Experiences

**DOI:** 10.1371/journal.pone.0323125

**Published:** 2025-05-06

**Authors:** Shuo Fei

**Affiliations:** School of Public Policy and Management, Tsinghua University, Beijing City, China; Cardiff University, UNITED KINGDOM OF GREAT BRITAIN AND NORTHERN IRELAND

## Abstract

This paper investigates the implementation of personalised support under Universal Credit (UC) from both administrative and claimant perspectives, based on qualitative interviews conducted across two South London case study sites. While UC is designed to promote labour market participation and simplify welfare delivery, claimants encounter multiple barriers—including digital exclusion, limited access to affordable childcare, and inconsistencies in support provision. Parents with care responsibilities face heightened pressures due to tightened work requirements and upfront childcare costs, which are often regarded as limiting their ability to comply with conditionality. Local frontline staff navigate institutional pressures from multiple governance levels within a context of resource constraints, applying discretion to tailor support to individual needs. However, this discretion may result in variable practices and uneven outcomes across different service settings. The findings underscore the tensions between the policy goal of responsibilising individuals and the complex realities of claimants’ lives.

## 1. Introduction

Welfare-to-work, positioned at the critical nexus of neoliberalism and state paternalism, serves as a valuable analytical lens through which contemporary governance and the reform of social policy may be comprehensively examined. This paper provides a critical interrogation of the dynamic interplay between neoliberal ideology and paternalistic modes of governance, with particular emphasis on market-oriented solutions and the promotion of individual responsibility, and explores how these forces shape welfare policy implementation and influence behavioural change among recipients.

Under the neoliberal paradigm, the concept of the ‘entrepreneurial self’—an individual striving for self-sufficiency within a competitive market—is deemed normative rather than ontological [1]. This creates a conflict between the institutionalised notion of rational entrepreneurship—characterised by deliberate calculation, cost-benefit assessment, and an emphasis on efficiency—and behaviours shaped by social, moral, and emotional considerations [1,2]. Such an ideal often contrasts with the lived experiences of welfare recipients, who grapple with systemic barriers incongruent with market-centric values. The varied implementation forms of neoliberal policies, characterised by privatisation and stringent conditionalities, frequently fail to address these deeper social issues, resulting in measures that appear more punitive than empowering [3,4].

This paper examines personalised support for Universal Credit (UC) from both administrative and claimant perspectives. Utilising an embedded case study analysis, this study challenges the prevailing discourse on welfare conditionality and behaviour change, foregrounding the voices and experiences of benefit claimants. It critiques the institutionalised narratives that often govern paternalistic interventions, highlighting the potential for personalised approaches that underpin the autonomy and dignity of individuals, considering their interconnected and complex individual circumstances. In light of Zacka’s view [5], the personalised approach should move beyond the confines of neoliberal reductionism, advocating for policy implementations that are accessible, responsible, and equitable, and attuned to the socio-economic complexities surrounding welfare recipients.

Furthermore, this paper examines the perspectives of UC claimants and administrative staff on personalised support, providing broader insights into the implications of neoliberal frameworks on the delivery of welfare services. It reveals the complexities and challenges faced by both claimants and practitioners, highlighting the inconsistent nature of personalised support within a resource-constrained environment. By examining the nuanced experiences of individuals engaging with the Universal Credit system, this study offers critical insights into welfare reforms that seek to responsibilise claimants through locally administered support mechanisms. It provides broader lessons for understanding the governance of welfare-to-work programmes, highlighting the imperative for policies that reconcile neoliberal imperatives of efficiency and administrative rationality with principles of fairness, responsiveness, and holistic support.

## 2. Empirical Case: Universal Credit

### 2.1 Access of Universal Credit

Universal Credit (UC) was introduced with the intention of simplifying the welfare system and reducing bureaucratic complexity. As the Conservative Party noted [6], the cumulative cost of navigating successive regulations across the public, private, and voluntary sectors was seen as stifling innovation, civic engagement, and social responsibility. The broader trend towards the digitisation of social policy and public administration has reconfigured citizen-state interactions, underpinned by the assumption that services can be delivered through automated, default mechanisms—such as ‘social web’ behaviours, zero-touch digital platforms, personal online accounts (‘journals’), self-service functionalities, and co-production models [7]. However, this digital-by-default approach poses significant challenges for many claimants, particularly those without adequate digital skills or access to online resources. These difficulties extend to navigating available support mechanisms, including assisted digital support and personal budgeting support [8].

UC replaces six social security benefits (Income-Based Jobseeker’s Allowance, Income-Based Employment and Support Allowance, Income Support, Housing Benefit, Child Tax Credit, and Working Tax Credit) with a single means-tested benefit [9] that reflects the feature of ‘reintegration’ of digital-era governance [10]. A further distinctive aspect of recent social policy reform lies in UC’s integration of in-work and out-of-work benefits into a single payment system, purportedly to strengthen behavioural conditionality by promoting continuous engagement with the labour market.

UC has been portrayed as a way of simplifying the administration of the benefits system, moving recipients off benefits and into the paid labour market, and reducing overpayments due to fraud and error [11]. The process for claiming UC aims to achieve frontline simplicity through automation [12]. UC applications are submitted online, requiring claimants to supply a range of personal and financial information. This includes bank account details, an email address, access to a telephone, National Insurance numbers, housing circumstances (such as rental costs), employment and income records, savings and investments, health and childcare arrangements, as well as information on any other benefits currently received [11]. However, this administrative view of simplicity does not necessarily achieve simplicity from the perspective of claimants and frontline stakeholders in terms of accessing and understanding UC [12]. During the COVID-19 pandemic, the absence of face-to-face support services in certain areas disproportionately affected specific claimant groups—such as individuals for whom English is not a first language, members of BAME communities, people experiencing homelessness, asylum seekers, and those with limited digital literacy—who encountered significant barriers in accessing the necessary technology to engage with the online UC system [13].

New claimants of UC typically receive their first payment after a period of approximately six weeks. This timeframe includes seven initial waiting days following the claim, a one-month assessment period, and an additional week for calculating entitlement and issuing the award [11]. Since late 2017, this initial waiting period has been shortened to five weeks [11]. UC is paid in arrears, with earnings assessed over the month following the claim, and payment issued seven days after the assessment period concludes [11]. Nonetheless, many claimants experience significant delays in receiving their first payment, often resulting in financial hardship—particularly for those previously accustomed to receiving more frequent payments under the legacy benefit system [12]. The transition to a single monthly payment structure can also complicate household budgeting, especially for individuals who formerly received different benefits at staggered intervals [14]. For claimants who experience changes in their monthly income, the monthly assessment period of UC is experienced as inflexible, which can lead to overpayments or underpayments.

Claimants have the option to request an advance payment prior to receiving their first Universal Credit (UC) award [15]. In 2017, these advances were typically issued within a week, with some claimants receiving the payment as early as three days before their first scheduled UC payment [11]. The amount received through an advance is subsequently recovered through deductions from future UC entitlements [11]. Furthermore, claimants who encounter difficulties managing the standard UC payment structure may apply for an Alternative Payment Arrangement (APA). APAs are designed to offer greater flexibility by enabling more frequent payments, facilitating direct payment of the housing element to landlords (referred to as a ‘managed payment’), and allowing the household payment to be divided between partners in a couple [16]. APAs can assist claimants in achieving financial independence. However, determining eligibility for APAs requires accommodating the complexities of claimants’ lives to ensure those who do not fit into predefined vulnerable groups do not slip through the net [17]. Furthermore, collaboration between landlords, Jobcentre Plus (JCP), Local Authorities (LAs), and other local services can leverage their knowledge and data to provide a comprehensive understanding of claimants’ situations, clarify what evidence can be used to make decisions around APAs, and ensure consistent application of policy. This involves (re)training and resources for landlords to proactively identify applicants who may need APAs. The system should be flexible and responsive to changes in claimants’ circumstances, allowing for necessary adjustments to APAs as needed [17].

### 2.2 Conditionality and Sanction

Universal Credit (UC) is designed to address issues related to low income, low-waged, and often insecure employment. UC is intended to promote labour market participation by incentivising individuals to enter paid employment, reducing reliance on welfare, and ensuring that work is consistently financially advantageous [18]. From a radical perspective, UC seeks to change values and behaviour within the benefits system [14]. UC is designed to mimic employment, fostering ‘independence and personal responsibility’ among claimants [19]. Claiming UC is akin to fulfilling an employment contract, with unemployed applicants moving into paid work while receiving benefits and support services. The government, in this case, functions as the claimants’ employer, intentionally replicating an employment contract. The claimant commitment emphasizes that receiving benefits is essentially equivalent to being employed [11]. Another aspect of transforming culture and behaviour is that UC is paid directly and monthly to mimic wages. In line with the broader extension of market logic and financialisation, UC consolidates six separate benefits and tax credits into a single payment. Claimants are expected to assume greater responsibility for managing their finances and budgeting behaviour, both while in employment and during periods of unemployment.

Shaping behaviour and values is central to the emphasis on conditionality and benefit sanctions, with the aim of strengthening ties to the formal labour market through work requirements. UC broadens the scope of conditionality by extending it beyond out-of-work claimants to include those in low-paid, part-time, and insecure forms of employment, such as zero-hour contracts. These individuals may be subject to sanctions if they fail to comply with the stipulated requirements [20]. This implies that both non-working and working claimants who do not earn enough to meet their conditionality threshold are subjected to personalised, extended, and intensified conditionalities. Conditionality has been characterised as contractualist, paternalistic, and utilitarian in nature, functioning as a policy mechanism aimed at encouraging citizens to assume personal responsibility, succeed through employment, counter irresponsible behaviour, and refrain from engaging in actions deemed anti-social [21].

A central feature of Universal Credit’s (UC) personalised and increasingly intensive behavioural conditionality regime is the emphasis on the individual’s action plan and the Claimant Commitment (CC). These mechanisms are intended to steer behavioural change, particularly by incentivising sustained job search efforts and progression into paid employment [18]. The CC sets out detailed requirements, including job-search tasks and participation in mandatory training programmes such as the Work Programme, which claimants must fulfil to maintain benefit entitlement [22]. Claimants earning below the conditionality threshold—approximately equivalent to 35 hours per week at the national minimum wage—are expected to increase their earnings or working hours accordingly. Significantly, UC does not impose an upper limit on the number of hours claimants may work [11]. Those in receipt of UC while employed are also expected to demonstrate in-work progression by pursuing additional or higher-paying roles [15]. The CC further stipulates that non-compliance without a valid, officially recognised reason—termed ‘good cause’—may result in sanctions [22]. This approach reflects an underlying assumption that individuals possess both the responsibility and the capacity to prepare for, access, and advance in the labour market [19].

According to the Department for Work and Pensions [23], a benefit sanction is applied when ‘a claimant fails to meet any of the responsibilities agreed in the Claimant Commitment without good reason,’ resulting in a reduction of their benefit payment. The strengthened sanction regime, introduced in 2012, established a four-tier system—comprising lowest, low, medium, and high levels—through which penalties are imposed on claimants who do not fulfil the work-related requirements outlined in their CC (see Table 1). The most severe of these measures, the three-year sanction, was abolished in 2019.

**Table 1:** Enhanced Sanction Regime Introduced Since 2012 prior to UC.

Sanction Level (for UC and JSA claimants)	Length of Sanction		
	First Failure (within a 52 week period of their last failure)	Second Failure (within a year within a 52 week period of their last failure)	Third and subsequent failure (within a year within a 52 week period of their last failure)
High Level	13 weeks	26 weeks	156 weeks
Intermediate Level	4 weeks	13 weeks	13 weeks
Low level	Until claimant complies plus 1 week	Until claimant complies plus 2 weeks	Until claimant complies plus 4 weeks
Lowest Level	Until claimant complies	Until claimant complies	Until claimant complies

Source: DWP [23]

Benefit sanctions have faced considerable criticism for their relative severity, particularly in relation to vulnerable claimants, including those with physical and mental health conditions. Many within these groups have struggled to navigate the system and to meet the obligations set out in the Claimant Commitment (CC) [24]. Since October 2012, sanctions have been used intensively [20]. Benefit sanctions disproportionately affect lone parents (previously subject to light conditionality), and young people aged below 25 years [20]. The number of Jobseeker’s Allowance (JSA) claimants subjected to sanctions rose markedly after 2008, reaching a peak of 899,960 in 2013. This figure represents an increase of approximately 150,000 compared to 2011. Following the implementation of the revised sanction regime, the number of sanctioned claimants in 2014 was twice that recorded in 2009. These developments have prompted systematic critiques on multiple fronts, notably by Reeves and Loopstra [25] and Webster [26].

The official definition of ‘good cause’—used to determine whether a claimant’s behaviour constitutes compliance or non-compliance—has been criticised for being overly narrow and detached from the realities faced by many claimants [27]. Claimants who have been sanctioned may apply for a hardship payment, a discretionary form of support intended to cover essential living needs. To qualify for such payments, UC claimants are typically required to demonstrate that specific conditions have been met, including efforts to avoid spending on non-essential items and full compliance with work-related requirements [23]. These payments are issued as loans, which must be repaid once the sanction period ends [23].

Webster’s [28] report reveals that only one in four sanctioned JSA claimants actually received hardship payments. Oakley’s [29] review similarly highlights the significant barriers to accessing such payments, raising concerns about claimants’ interactions with, and understanding of, the system. A further contentious issue associated with sanctions is the growing dependence of claimants on food banks and charitable support [30]. Quantitative research by Loopstra et al. [31] demonstrates a correlation between rising sanction rates and an increase in ‘welfare exits’, though it also points to a disconnection between welfare withdrawal and sustainable employment. Their findings suggest that while sanctions may prompt claimants to leave the benefits system, they do not significantly contribute to employment recovery. However, this study offers limited insight into the qualitative dimensions of behavioural change. Grover [32] critiques this by arguing that sanctions do not necessarily result in meaningful changes in employment behaviour. In a more comprehensive analysis, Wright et al. [33] emphasise that sanctions tend to intensify financial hardship, indebtedness, and anti-social behaviours. They further argue that sanctions adversely affect claimants’ mental health and impose additional, unnecessary obstacles to securing paid employment.

Reforms to conditionality and sanctions can be understood through the lens of Foucault’s [34] concept of neoliberal governmentality, which operates through mechanisms of vigilance, activity, and intervention. These reforms seek to produce active and productive citizens by institutionalising behaviour change within a framework that moves away from a traditional, state-centric model of welfare provision [25,34]. Concerns regarding both the effectiveness and the ethical legitimacy of such approaches have been raised by numerous scholars [25,35–38]. Drawing on Wacquant’s [39] theoretical framework, Fletcher and Wright [37] argue that the expansion and intensification of conditionality can be critically analysed as part of broader punitive shifts in welfare governance. Far from encouraging engagement, welfare conditionality often alienates claimants from both social support systems and the labour market, thereby compounding financial hardship, mental health difficulties, and poverty [4].

One of the most counterproductive aspects of conditionality lies in the unrealistic expectations embedded within the Claimant Commitment (CC), which frequently overlook the complexities of claimants’ individual capabilities, responsibilities, and vulnerabilities. These include difficulties in complying with mandated activities such as using the Universal Jobmatch system, attending training programmes, or participating in mandatory interviews [33]. Oakley’s [40] report highlights that a wide range of factors beyond active job searching—such as qualifications, mental and physical health conditions, recovery trajectories, age, and duration of unemployment—also shape employment outcomes. To foster more effective employment behaviours, scholars advocate for services that are supportive, less punitive, and grounded in understanding rather than enforcement [8].

The conditionality requirements for single parents receiving welfare support have progressively tightened since 2008. Before this shift, single parents were not obligated to seek work until their youngest child reached the age of 16. The age threshold for work requirements was initially lowered to when the youngest child turned 12, and by 2017, it had been gradually reduced to when the youngest child reached the age of three. This reflects a broader policy trend toward encouraging parental workforce participation [41].

The most recent and significant change to these rules came in October 2023, when the government introduced new work requirements for lead carers of children aged 3–12. Under the revised policy, parents with a youngest child aged three are now expected to be available for work or actively seeking employment for up to 30 hours per week. This represents a sharp increase from the previous requirement of 16 hours per week for parents with children aged three to four and from 25 hours for parents with children aged five to 12 [42]. Although the intention behind these changes is to increase labour market participation among parents, they have imposed significant challenges on low-income families. Many parents face difficulties securing affordable and accessible childcare, particularly since reimbursement for childcare costs through Universal Credit (UC) remains complex and insufficient. Parents must cover childcare costs upfront and then reclaim them through the UC system, which is subject to a 55% taper rate applied to earnings above the work allowance. This means that many parents do not receive the full amount of childcare costs they incur, adding financial strain to already vulnerable households [42].

Furthermore, the increased conditionality has disrupted the work-care balance for many families. Parents, particularly those in low-income households, have reported that available childcare hours often fall short of their actual needs. Childcare providers may also charge extra fees for meals and administrative costs, which are not covered under the UC system. Parents of children with disabilities or additional needs face even greater barriers in securing appropriate care. The increased work requirements have thus introduced not only financial pressure but also difficulties in arranging adequate childcare, coordinating work schedules, and accessing suitable care options for many families [42].

### 2.3 Carer’s Element of Universal Credit

The Carer’s Element of Universal Credit aims to mitigate the financial stress of carers who support the healthcare system and ensure the well-being of individuals with disabilities [43]. It partially compensates for the economic value of unpaid care work, which is usually undervalued in the labour market, by providing £198.31 per month for eligible carers [44]. To be eligible to claim this element, the carer must be eligible for Universal Credit and must not have claimed other benefits to cover similar needs, such as Carer’s Allowance. The carer must provide care work for at least 35 hours per week for a person who receives a qualifying disability benefit, such as the Disability Living Allowance (DLA), Personal Independence Payment (PIP), or Attendance Allowance [43]. The Carer’s Element has broader implications for reducing gender disparities by recognising unpaid labour, which is predominantly undertaken by women. In comparison, Income-Based Jobseeker’s Allowance and Carer’s Allowance had separate rules of eligibility, creating challenges for claimants who had to balance job search commitments with significant caregiving responsibilities (see more discussion in section 4.1).

### 2.4 Nature and Governance of UC Support Services

Universal Credit (UC) is administered by the Department for Work and Pensions (DWP), marking a departure from the legacy benefits system, in which tax credits were managed by HM Revenue and Customs (HMRC), while the DWP oversaw most means-tested benefits. The implementation of UC reflects a more integrated model of governance, drawing on both Weberian bureaucratic principles and the evolving post-New Public Management (NPM) paradigm [10]. UC governance represents a dynamic interplay between centralised policymaking and decentralised local delivery.

The delivery of UC—branded as ‘Universal Support’ or ‘Help to Claim’—is predicated on the assumption that managerial and frontline actors within local authorities and the third sector will assist claimants in navigating the administrative and digital complexities of the system [13]. ‘Help to Claim’ services include Assisted Digital (AD) support and Personal Budgeting Support (PBS), both of which are aimed at improving digital engagement and financial capability [11]. Under this model, central government works in partnership with local stakeholders to identify context-specific needs and deliver a range of support services—such as online claims assistance, financial advice, and job search guidance—designed to foster long-term claimant self-sufficiency [11]. The decentralised nature of UC implementation entrusts significant decision-making responsibilities to local actors, including local authorities, Citizens Advice, credit unions, housing associations, and charitable organisations [11].

To support this governance architecture, the DWP has invested in a framework of assistance aimed at enabling individuals to build more secure futures for themselves and their families, while regaining a sense of autonomy and control [45]. UC plays a central role in this agenda by seeking to shift claimants from welfare dependency to economic independence [15]. Emphasising employability, UC is structured to facilitate a smoother transition from unemployment to paid work and to ensure that employment offers clear financial advantages over remaining on benefits [15]. A key mechanism in this regard is the single taper rate, under which benefit entitlements are gradually reduced as earnings increase. This design feature is intended to guarantee that claimants always derive financial gains from increasing their work hours.

However, evidence suggests that this goal is not always realised in practice. Wood et al. [42] highlight that the childcare costs reimbursement under UC creates financial strain for many parents, as they are required to pay costs upfront and then reclaim them through the system. Even though UC covers up to 85% of childcare costs, the reimbursement is capped at £1,015 per month for one child and £1,739 for two or more children, which often does not fully cover the actual expenses [42]. Moreover, the amount parents receive fluctuates due to the 55% taper rate applied to earnings above the work allowance, which reduces the overall UC payment, including the childcare element [42]. This tapering effect, combined with the monthly assessment period, results in delayed and inconsistent payments, making it difficult for claimants to budget effectively [42]. Furthermore, the absence of a second-earner work allowance discourages additional work, particularly for female partners in couple households, as any increase in household income can lead to a reduction in other means-tested support, such as Council Tax Reduction and free school meals, leaving families worse off despite working more hours [42].

Personalised support from work coaches constitutes a core element of UC’s employability strategy. Upon initiating a claim, individuals are assigned a dedicated work coach (or adviser) through Jobcentre Plus (JCP) [22,23]. These coaches are responsible for outlining the claimant’s job-search requirements and mandatory training obligations—such as participation in the Work Programme—that must be fulfilled to maintain eligibility for UC payments [18]. In addition to enforcing conditionality, work coaches provide tailored guidance to help claimants pursue both employment and health-related goals, including in-work progression. This support is designed to be responsive to each individual’s specific circumstances, capabilities, and barriers to work [23]. Another crucial aspect of the UC support programmes is empowering individuals through tailored professional needs and personal development initiatives [23]. These programmes include personalised job coaching, training, and support services that address individual barriers to paid employment and help claimants navigate the complexities of the labour market. Eligible claimants can apply for up to 85% of childcare costs [23]. The aim is to equip claimants, including those from BAME backgrounds and women, not only with vocational skills but also with the agency to move into paid labour, close the lone parent employment gap, and further increase female employment rates. Concerns have been raised about ‘creaming’ and ‘parking’ in contracted-out employment services, where individuals perceived to have the highest likelihood of moving into paid work are given priority, while those who are harder to help are ‘parked’ within limited resources [46].

Frontline stakeholders in local state and third sectors transform policies into regional and local practices, especially when policies are ambiguous or inconsistent [47]. They mediate politics when making decisions or interpretations at the front level [48]. They often perform dual control when assisting claimants [49]. They exercise discretionary power based on their professional judgement and the specific needs of claimants. For instance, work coaches at Jobcentre Plus (JCP) exercise both control and conciliatory functions to ensure that claimants are sufficiently motivated to meet their conditionality requirements. As demonstrated in this paper, frontline stakeholders frequently engage with claimants’ individual needs, offering professional support that respects their autonomy, preferences, interests, and overall wellbeing. This role requires frontline actors to navigate and reconcile conflicting problem definitions and balance competing organisational objectives within complex institutional settings. Frontline work is structured since frontline workers operate in complex contexts that may constrain discretionary practices and limit their decision-making [47]. Within this specific context, the availability of financial and administrative resources to support claimants varies considerably across local areas and tiers of governance. These disparities are shaped by broader structural pressures, including the ongoing political climate of austerity, the impact of the COVID-19 pandemic, and rising demand for support services [50,51]. This requires organizations to exercise more discretion in managing resources and building networks to support referrals and address community needs. It also necessitates that local actors balance conflicting priorities within organizational environments [13]. Moreover, disparities exist in how effectively local support services can offset the reductions in working-age social security for households and regions with the lowest incomes [50]. These differences are linked to the extent, sufficiency, and availability of local services, as well as variations in service quality [24].

## 3. Research Design

This study adopts an embedded case study design, which is particularly well-suited to the qualitative exploration of behaviour change in context-specific and contingent settings. The case study approach enables the development of what Weber [52] referred to as an immersive understanding of participants’ experiences, and what Schutz [53] termed intersubjectivity between researcher and participant. It facilitates rich, nuanced descriptions and critical interpretations grounded in participants’ lived realities [54]. Such understandings are shaped through dynamic interactions between researcher and participant, as well as through participants’ own meaning-making processes informed by social experience [54].

To enhance the analytical depth and generalisability of findings, this study adopted a multi-level embedded design across three units of analysis: (1) two distinct geographical locations in South London; (2) five categories of local institutions—Local Authorities, Advice Centres, Housing Associations, Jobcentre Plus (JCP), and Food Banks; and (3) individual participants, either employed within these organisations or receiving Universal Credit (UC).

A purposive sampling strategy was employed to recruit individuals with direct experience of UC, either as claimants or professionals involved in its implementation. The final sample comprised 32 claimants of working-age social security, including both in-work and out-of-work recipients, and 18 stakeholders from the aforementioned organisations (see Tables 2 and 3). To maximise diversity and contextual richness, non-probability sampling techniques—including snowball and convenience sampling—were also utilised. These methods enabled the inclusion of participants with varied social, institutional, and digital access experiences, thereby generating a heterogeneous dataset.

**Table 2:** Study population – stakeholders.

Institutions and Advice Agencies	Study Site and the Number of Respondents
**1. Local Authorities**	7
Interim Head of Revenues, Benefits, Insurance and Pensions	A: 1
Welfare Reform Outreach/Enablement Manager	A: 1; B:2
Employment Support Manager	B:1
Discretionary Housing Payments and Discretionary Scheme manager	B: 1
Housing Benefit team staff	B:1
**2. Advice Centres**	9
Welfare Support Team Manager/ Benefit and Debt Advisor in Housing Associations	A: 2; B: 1
Work Club, Debt Advice officer/Coach in Charities	A:3; B:3
**3. Food Bank**	2
Food Bank volunteers	B:2

Source: Author’s research data.

**Table 3:** Study population – claimants.

Category	Detail	Count/ Description
**Gender**	Male	15
	Female	17
**Household composition**	Single jobseekers	13
	Lone parents with dependent children	10
	Couples without dependent children	5
	Couple with dependent children	4
**Age Structure**	20–25	1
	26–49	21
	50–65	10
**Ethnicity**	White British	16
	African/ Caribbean British	6
	Asian British	1
	European citizens	3
	African/ Caribbean origins	6
**Employment Status**	In-work	14
	‘Mini’ jobs	7
	Full-time jobs	5
	Self-employed	2
	Out-of-work	18
**Tenure**	Owned house	8
	Private rented	19
	Social rented (excluding LA rented)	3
	LA rented	2
**Industry of Employment History**	Administrative & support services	7
	Financial & insurance activities	2
	Construction	5
	Long-term unemployed	2
	Wholesale, retail & repair of motor vehicles	9
	Professional, scientific & technical activities	1
	Transport & storage	1
	Public admin & defence; social security	2
	Accommodation & food services	3
**Working age benefit claiming duration**	Less than 1 year	2
	1–5 years	17
	5–10 years	8
	More than 10 years	5
**UC claiming duration**	3–6 months	7
	6 months to 1 year	7
	More than 1 year	12

Source: Author’s research data.

To capture these experiences across different local welfare ecosystems, two field sites were purposively selected. Site A, referred to as ‘Urban Settlement’, and Site B, ‘Ethnically Diverse Metropolitan Living’ [55], differed significantly in both institutional infrastructure and demographic profile. According to the 2021 Census, ethnic minority populations constituted 31.7% in Site A and 51.6% in Site B [56]. Both sites had implemented the full UC digital service by April 2016, rendering them suitable for examining the digital dimension of welfare access and behavioural conditionality.

Institutional arrangements also varied across the two sites. For example, Site A had only one JCP office and one advice centre providing Universal Support, with a single voluntary Food Bank operating across two locations. In contrast, Site B afforded broader access to support services and stakeholders, allowing for richer insights into institutional interaction and the co-production of behavioural change. Notably, Site A claimants frequently presented with mental or physical vulnerabilities, while Site B participants more commonly reported digital or literacy-related barriers, which may be linked to its larger population of residents whose first language is not English [55].

Participant recruitment was conducted between 1 October and 30 November 2017. Initial outreach involved postal correspondence to stakeholders within JCPs, Local Authorities, and advice centres, enclosing approved cover letters and participant information sheets. Due to restrictions imposed by the Department for Work and Pensions (DWP), direct interviews with work coaches were prohibited; hence, recruitment was supported by managerial and front-line staff from community organisations, who distributed invitations to potential participants. Recruitment was further supplemented through online forums and UC-related Facebook groups, with all communications maintaining anonymity and confidentiality.

The study received ethical approval from the Research Ethics Sub-Committee of the University of Nottingham under reference number 127-16-17-PGR. Informed consent was obtained from all participants, who were fully briefed on the study’s aims, procedures, risks, and their right to withdraw without penalty. Written consent was secured prior to participation, and all names used in this thesis are pseudonyms to ensure anonymity and uphold ethical standards.

All interviews were transcribed and analysed using *NVivo 12*. Field notes were also taken during the data collection process to capture contextual observations. During the initial phase of data analysis, transcripts were read and re-read to allow for immersion in the data. Descriptive nodes were first developed from the initial five interview transcripts, forming the basis for a preliminary coding framework. This framework identified emerging themes such as the ‘barriers’ and ‘bridges’ encountered in the process and outcomes of behavioural change. These initial nodes, and coding frame were reviewed and changed, as the rest interview transcripts were reviewed and coded via open coding. Selective coding was undertaken to develop analytical dimensions that captured stakeholders’ and claimants’ experiences and perceptions. This process facilitated a deeper understanding of the underlying dynamics associated with benefit claiming, reporting changes in circumstances, and employment-related behavioural change.

For in-depth analysis, the relationship between the thematic coding framework and the research questions was systematically examined and refined. This involved clustering related codes to generate higher-order themes and identifying key factors that shaped the processes under investigation. The thematic coding framework was also mapped onto theoretical concepts related to behaviour change, with particular attention given to the compatibility—drawing on Weber’s notion of ‘Verstehen’—between sub-concepts within the coding and the analytical framework of administrative burdens [57]. Through axial coding, sub-concepts were allocated to broader thematic categories in relation to administrative burdens, enabling more nuanced interpretations of the data. This process contributed to iterative scrutiny, refinement, and eventual saturation of themes as the analysis progressed [57].

Employing the concept of administrative burdens as an analytical lens enabled an exploration of how claimants made sense of policies that affect their autonomy, competence, and sense of relatedness. This applied to both the formal implementation of means-tested and conditionality-based social policies and the informal, non-legislative practices enacted by frontline stakeholders.

Both within-case and cross-case analyses were conducted. Within-case analysis entailed detailed, descriptive examination of individual participants’ interpretations of their benefit and employment experiences [54]. Thick description was used to explore the context, motivations, attitudes, strategies, and intentions of each case, with the aim of assessing the extent to which specific cases reflected patterns that could be generalised [54]. Cross-case analysis was employed to identify relational patterns between individual respondents and the broader sample. A matrix coding technique was used to explore associations between participants’ attributes (e.g., age, gender, ethnicity, family composition, location) and key themes (e.g., benefit claiming and employment-related behaviours), with colour shading used to visually represent the intensity or presence of these associations in each cell. These matrix query results were incorporated into the explanatory case study analysis and contributed to identifying both convergence and divergence across cases, supporting broader interpretive generalisations [54].

## 4. Empirical Findings

### 4.1 Recognition of Caregiving

Pre-UC, the separation of Jobseeker’s Allowance (JSA) and Carer’s Allowance created rigid policy conditions that overlooked the complex realities faced by claimants. Individuals with substantial caregiving duties, particularly women, frequently found themselves caught between conflicting obligations: meeting strict job-search requirements or fulfilling intensive unpaid caregiving responsibilities. Anna’s experience vividly illustrates these tensions. Due to her significant caregiving duties for a disabled person at home, Anna (pseudonym) struggled to meet the rigid conditionality requirements of JSA, resulting in the risk of losing financial support. She explained:

They [IB-JSA system] were not understanding of situational difficulties; it was either I looked for work or I [did caring responsibility for a disabled person at home and hence] was kicked off the whole JSA. They [DWP staff] did not understand how everything was going at home. So, the programme [IB-JSA] did not take [the caring responsibility for a disabled person] into the assessment. When I was a carer [for a disabled person], they would give me carer allowance and I wouldn’t be on IB-JSA, or whatever it is. This means for IB-JSA, they did not take into consideration the fact that [caring] things can go on, that [caring responsibility] can impact my job searching, so UC does [include the caring responsibility into] changes circumstances at home. With the pressure being lifted, I can do more [towards fulfilling work search commitments]. (Anna, aged 29, female, single, African/Caribbean British, site A)

Under UC, Anna can now qualify for the Carer element while simultaneously managing her caregiving and work-related commitments. By integrating caregiving responsibilities into eligibility and conditionality assessments, UC represents a significant policy advancement compared to the pre-UC system. This integrated approach alleviates the tension previously experienced by claimants like Anna, enabling them to navigate both caregiving duties and employment requirements without risking the loss of vital financial support. Crucially, UC’s recognition of caregiving roles reflects a broader shift towards more inclusive and responsive social welfare policies, with positive implications for women’s labour market participation and their ability to secure adequate resources for paid domestic care.

However, the recognition of caregiving under UC remains inconsistent, particularly regarding childcare, work requirements, and financial support. Beth’s (pseudonym) case highlights this contradiction. Although Beth is formally exempt from work requirements due to having a six-month-old child, she reported experiencing implicit pressure to seek employment. Beth explained: ‘UC [work coach] contact me all the time, trying to get me back into work, because they want people in work.’ This indicates that while the system formally exempts parents with young children from explicit work-related conditionality, it nonetheless creates an implicit expectation of employment through ongoing interventions by work coaches.

Beth’s difficulty accessing the 30 hours of free childcare underscores a significant limitation within childcare eligibility regulations. According to government policy, both parents must be in employment, each earning at least the equivalent of 16 hours per week at the National Minimum Wage or National Living Wage. Beth’s partner is not employed and did not meet residency requirements for UC, which prevented their household from qualifying for the 30 hours of free childcare, despite Beth herself potentially being eligible if assessed independently. Additionally, Beth faces considerable financial strain due to UC’s childcare payment model. She explained: ‘I just cannot afford it.’ Beth must initially cover £800 per week in childcare costs herself, with UC reimbursing up to 85% afterward. This ‘pay first, claim later’ system creates substantial cash flow challenges for low-income households, highlighting a critical gap in the practical support provided by UC.

Clara’s case further illustrates the challenges within UC’s approach to childcare and work incentives. Clara’s work coach advised her that this part-time arrangement was insufficient and encouraged her to seek 32–38 hours of work per week. Clara described the practical conflict this created: ‘I cannot start from 8 a.m. because I have to send my son to school.’ This reflects a disconnect between the working hours required to meet UC’s conditionality and the limited availability of affordable childcare that fits around school hours. School hours typically run from around 8:30 a.m. to 3:30 p.m., creating a gap between working hours and childcare availability. Many jobs that Clara could access require early morning or evening shifts, which are incompatible with the school schedule. Without access to flexible and affordable childcare outside of school hours, it can be challenging for Clara to balance UC’s work expectations with her caregiving responsibilities.

Clara also highlighted the instability created by zero-hours contracts: ‘zero hours contract is more flexible, but not enough to cover my expenses.’ While zero-hours contracts provide flexibility in terms of scheduling, the unpredictable hours and income make it difficult for Clara to budget and meet UC’s work requirements. The irregular income from zero-hours contracts compounds the financial strain created by childcare costs and the tapering of UC payments as earnings increase.

### 4.2 Access to UC

Frontline stakeholders from both study sites consistently highlight significant access barriers faced by vulnerable individuals in navigating UC’s digital claiming processes and maintaining continuous access:

It’s all online, and it can be very difficult for some people [who are] trying to maintain the claim. (Warren [pseudonym], housing benefit team frontline staff, site B)

Stakeholders from Local Authority site A further emphasise the substantial digital exclusion among their clients, directly obstructing access to UC services: ‘my customers, I can say 80%, can’t [access digital claiming]’ (Jack [pseudonym], welfare reform outreach manager, site A). Claimants’ experiences similarly illustrate these digital barriers, highlighting the impact of limited digital literacy and resources on their ability to engage effectively with UC: ‘it’s difficult because not everyone has the internet or understands how to use computers. I had to get help every time I logged into my account because I didn’t know how to manage it.’ (Ruby [pseudonym], out-of-work lone parent, African-Caribbean origin, Site B)

Further complexities stem from the structure of UC itself. Stakeholders note that multiple benefit elements within a single UC claim often increase the likelihood of administrative errors: ‘the more elements somebody has, the more transactions there are for UC to get it wrong’ (Alicia [pseudonym], in-house benefits and debt advisor, Housing Association, site B).

Given the demographic context of site B, with its high proportion of residents from minority ethnic backgrounds whose first language is not English, digital and literacy barriers disproportionately limit access to UC services: ‘site B is a large borough, with a high level of immigration, so there are going to be many people who need a lot of support’ (Kay [pseudonym], welfare rights team manager, site B).

Access is further undermined by discrepancies in UC’s reimbursement model for childcare costs. As noted by Judy (employment support, Site B):

If you want to place your child in nursery, you’ve got to be careful—nursery fees are paid in advance, but UC only pays in arrears. It’s a bit like the waiting days—only so much is covered. So anybody who has a child in nursery struggles with the UC childcare element being paid directly to the nursery. It’s not working.

Such delay in childcare reimbursements, place low-income families under considerable financial strain and often prevent sustained access to work-related childcare support. In this context, community and third-sector organisations have become vital actors in mitigating the effects of these systemic barriers, providing not only practical assistance with claims but also fostering claimants’ confidence and employment readiness. As Anna explained:

They [staff in the charitable body] helped me to claim UC and find a job actually. They told me about different websites that would directly go to the job vacancy, two weeks later I applied for it and then I got the interview for it.

### 4.3 Pressures to Meet Ongoing Needs

Both study sites face resource constraints that are perceived to have a considerable effect on their capacity to deliver sustained support to Universal Credit (UC) claimants. Claimants who require digital assistance frequently depend on external agencies, thus exacerbating pressures on local services already strained by limited resources. From an administrative stakeholder perspective, providing comprehensive social support is viewed as financially burdensome. Wendy (pseudonym), a welfare support team manager at a social housing association in Site A, articulated the consequences of funding cuts on service provision, reflecting the broader context of increased caseloads and extended responsibilities following the introduction of UC:

Our management fee is less and the services we were expected to provide before are no longer there because financially, the money isn’t there. We are always driven by the government and the local government. We deal with very complex cases, and UC recipients seem to visit us repeatedly, so we are taking on more responsibilities. It costs us more because we are doing more than we usually would, but in the longer term, it helps them sustain their tenancy and remain in their property.

Despite these financial and operational pressures, Wendy’s team continues to provide advice and support to UC recipients, focusing on sustaining their tenancies and helping them remain in their properties, even though this extends beyond their usual responsibilities and resources. However, the available support is often inadequate for addressing clients’ diverse and ongoing needs, revealing an intrinsic tension between immediate relief and longer-term independence.

Andy (pseudonym), a welfare support team officer in Site A, emphasizes a strategy aimed at fostering client autonomy: ‘eventually, people will have to learn to manage their money independently; unfortunately, we won’t always be able to do this for them.’ His viewpoint underscores a necessity for promoting self-sufficiency amidst resource scarcity, though this approach may inadvertently lead to inconsistent outcomes among clients facing similar hardships.

Moreover, dependence on short-term financial interventions, such as discretionary payments and emergency assistance, highlights the challenge of effectively addressing the sustained financial needs of claimants within existing resource constraints. Michael (pseudonym), Interim Head of Revenues, Benefits, Insurance, and Pensions at Site A, describes the repercussions of discontinued crisis loan funding: ‘we received crisis loan funding for two years, after which it ceased. We’ve since become much more stringent about the awards we give, as funding has been withdrawn.’ The stringent criteria applied to crisis loans due to funding constraints place additional strain on claimants who require immediate financial support. This situation risks leaving some individuals without necessary assistance.

At Site B, Carol (pseudonym), a discretionary housing payment team manager, outlines the limitations of temporary relief mechanisms: ‘they [clients] haven’t got any money, so they may come to us for our food bank referral. They might not be able to pay for gas and electricity, so again, we can help them pay that as a temporary measure,’ The temporary nature of this assistance underscores resource limitations faced by local services, indicating a gap between immediate financial relief measures and the ongoing, longer-term support needs of claimants.

In the context of growing institutional pressures, frontline services are increasingly constrained in their ability to meet the ongoing needs of claimants. Constrained staffing levels, combined with high service demand, lead to brief and transactional encounters, particularly disadvantaging claimants with complex needs:

I was informed by a member of LA staff that she could ‘only be with [me] for two minutes so [I had] to be quick, as [she had] three people waiting.’ She was very much in a rush. You can see that there is a long queue. Before [pre-UC], it’s [Local Authority’s service is] good because I can talk face-to-face, very good*.* (Jane, aged 55, Spanish, site B)

Jane’s (pseudonym) experience highlights how service delivery has become more transactional post-UC, reducing both time and relational quality in interactions. For claimants with limited digital or language skills, this shift undermines access and heightens the risk of disengagement. The move away from face-to-face contact thus reflects broader tensions between austerity-informed restructuring reform and the need for responsive, inclusive support.

### 4.4 Employment Support at Local Level

In Site B, local staff brought together several frontline teams—including welfare rights, housing, and employment support—within the same division. This arrangement was intended to support different aspects of claimants’ needs by enabling each team to contribute according to its own area of responsibility:

So we bring in main frontline services into one division. Within what we have the welfare rights team, housing options teams, public funds team, temporary accommodation teams, housing benefit, and council tax staff. We all work together to provide a realistic service to the individual or the family. (Kay [pseudonym], welfare rights team manager, Site B)

Rather than delivering the same services to everyone, staff used initial assessments to decide which type of help each person might need. This division of roles aimed to enable a more focused way of working, where claimants were referred to specific teams based on staff judgement. Claimants did not always refer to this structure explicitly, but some accounts showed how they encountered targeted forms of assistance through different channels.

One claimant, for example, recalled accessing a government-funded numeracy course that she found highly engaging: ‘it’s from the local community. It’s government sponsored. I stayed up until 3:00 am last night doing the maths work because I am so determined.’ (Danielle, out-of-work, Site B) Her comment points to how targeted support may be experienced as valuable when it aligns with claimants’ goals and self-perceptions of readiness.

Within the employment support team, staff used a combination of basic assessments and individual action plans to help claimants with financial and work-related concerns: ‘we work with each customer on an individual action plan based on a basic assessment, and then we give general advice about money saving, income maximisation, reducing debt.’ (Kay, Employment support team manager, Site B) This process was not described as offering a fixed sequence of interventions. Instead, it relied on staff deciding which issues to prioritise and what type of advice or referral to provide. The intention was to offer support that was practical and proportionate to the claimant’s situation. While claimants did not always refer directly to action plans, some spoke about receiving budgeting advice, or help understanding entitlements, often as part of a broader interaction.

Staff also supported job matching in some cases. This involved spending more time with clients and working with local employers: ‘we spend at least 60 minutes understanding your needs. Afterwards, we match you up individually with an employer here so that you can fit the employer to the employee.’ (Judy, Employment support team manager, Site B) Staff believed it could lead to a good employment outcome. Several accounts of claimants pointed to personal motivation and specific learning opportunities as meaningful steps toward employment, suggesting that such support—whether formally structured or encountered via other routes—could reinforce claimants’ sense of progress.

Claimants’ accounts also showed that employment-related support was accessed through various pathways, not all of which were clearly connected to local authority teams. Anna noted that the most useful employment help came from a third-sector organisation: ‘my advisor links me to several services, such as this Job Club. They helped me to build up confidence, with interview skills, and they do CV writing as well. They occasionally have people who go to the extra centre and help you with your interviews. So they helped with skills—the first step, step by step—the interviews, getting them into the job.’ Claimants’ experiences reflected this diversity of pathways: while some accessed assistance through local services, others engaged with publicly funded or community-based support without always linking it to the UC system. These accounts suggest that, in the current phase of UC implementation, employment-related support continues to be delivered through multiple institutional routes.

The fact that claimants engaged with employment-related services through various routes—such as local authorities, government-funded programmes, or third-sector organisations—reflects the presence of multiple, coexisting pathways into support. These pathways did not always follow a single formalised referral system, but were accessed through local knowledge, personal initiative, or community networks. While this pattern illustrates a level of adaptability within the UC environment, it also suggests that access to employment support may continue to depend on how services are organised and made available within local contexts. This highlights the value of understanding not only how services are delivered, but how they are encountered and acted upon by claimants within the wider institutional landscape.

## 5. Conclusion

The analysis of the Universal Credit (UC) system reveals critical insights into the dynamics of personalised support, illuminating the complex interplay between administrative practices and claimant experiences. This paper underscores the need for a more nuanced approach to service delivery—one that acknowledges institutional constraints while ensuring that claimants receive adequate support to pursue financial stability and independence. In doing so, it reflects and reinforces the neoliberal ideal of the self-reliant citizen.

From the claimant perspective, the UC system’s integration of six benefits/tax credits into one payment and its shift towards digital platforms reflect a neoliberal emphasis on streamlining government services to encourage individual responsibility and self-efficacy. Unlike the legacy benefits system, which had separate rules for different types of benefits, UC attempts to account for individual circumstances through a unified framework. This includes recognition of unpaid care work—especially for those caring for adults with disabilities—through mechanisms such as the Carer’s Element. However, the conditionality applied to parents with young children has intensified in recent years. While some parents are formally exempt from job-seeking requirements, others face rising expectations, with new rules requiring lead carers of children as young as three to engage in up to 30 hours of work-related activity per week. Claimants’ experiences in this study demonstrate the difficulty of meeting these expectations in the absence of accessible, affordable childcare. This tension suggests a growing policy disconnect between conditionality goals and the lived realities of caregiving, especially for low-income families.

For vulnerable adults, digital exclusion unwittingly creates new barriers. As Helsper and Reisdorf [58] highlight, digital exclusion disproportionately affects older adults, low-income households, and ethnic minorities. In the present paper, stakeholders view the digital divide and digital exclusion as factors that can hinder claimants’ ability to access and maintain their benefits, leading to unequal access and disproportionately affecting vulnerable groups, thus risking undermining the system’s fairness. Furthermore, the complexity of the UC claiming process, particularly for those with multiple benefit elements, may exacerbate these challenges. A report by the National Audit Office [59] demonstrates that the complexity and frequent changes in claimants’ circumstances can lead to errors and delays in payments. This can create a dependency on frontline staff to help navigate these processes, which may introduce variability and arbitrariness in how support is provided based on the availability and capacity of staff. This complexity can also be exacerbated for claimants with additional needs, increasing the risk of inconsistent support.

The resource constraints faced by local actors imply the neoliberal tendency towards market rationalisation. Difficult decisions often arise about which local residents, services, and activities should be prioritized. These compromises can vary across organizations and, within a single entity, between the policies it applies and the various stages of their implementation [60]. The evidence presented illustrates a tendency for the inconsistent and situational nature of personalised support to lead to situations where some claimants may receive more assistance than others, depending on where they seek support. Across both case study locations, the challenges of viability and funding constraints required local actors to balance standardized processes, broad guidance, and the demand for tailored support. The analysis highlights how local state and third sector professionals exercise significant professional autonomy and ethical discretion in responding to claimants’ individual needs. Yet such discretion may lead to inconsistencies and weakened accountability, particularly within the constraints of an austerity-oriented policy environment.

The evidence highlights that, facing resource reductions, local actors have to make difficult trade-offs about which services to prioritise and which clients to support. This scarcity can lead to decisions that appear arbitrary, as local actors may have to prioritise immediate needs over long-term solutions or vice versa. The risk of inconsistent application of discretion and the ambiguous status of personalised support can make claimants’ social rights and access to universal welfare vary depending on their needs and contingent situations. The competition for limited resources compels some local actors to adopt stringent criteria for support, and the necessity to ration resources can result in clients who require ongoing assistance facing a systemic push towards independence. These features shape state-citizen interactions and warrant further attention.

## Supporting information

S1 File Inclusivity-in-global-research-questionnaireSF.
(PDF)
