# Supplementary material for: Navigating Personalised Support in Universal Credit: Local Support, Care Responsibilities, and Claimant Experiences
Source: PLoS One. 2025 May 6;20(5):e0323125. doi: 10.1371/journal.pone.0323125 (PMC12054871; doi:10.1371/journal.pone.0323125)
Supplement: S1 File — Inclusivity-in-global-research-questionnaireSF. (PDF) [file pone.0323125.s001.pdf]

Inclusivity in global research

PLOS' policy on inclusivity in global research aims to improve transparency in the reporting of research performed outside of researchers' own country or community and ensures that PLOS publications reporting global research adhere to high standards for research ethics and authorship. Authors of relevant research articles may be asked to complete the questionnaire below, which outlines ethical, cultural, and scientific considerations specific to inclusivity in global research. This questionnaire may be requested when researchers have travelled to a different country to conduct research, if research uses samples collected in another country, research with Indigenous populations or their lands, or if research is on cultural artefacts. Researchers travelling to another country solely to use laboratory equipment will not normally be required to complete the questionnaire. However, the questionnaire can be requested at the journal's discretion for any submission – if you have been requested to complete this questionnaire by the PLOS journal you submitted to, please do so.

Please complete the questionnaire below and include this as a Supporting Information file with your manuscript. Note that if your paper is accepted for publication, this checklist will be published with your article in the supporting information files. Please ensure that you reference the checklist in the main body of your manuscript. We suggest adding a subsection 'Inclusivity in global research' to your Methods section and adding the following sentence: "Additional information regarding the ethical, cultural, and scientific considerations specific to inclusivity in global research is included in the Supporting Information (SX Checklist)"

The questions have been designed to be applicable to a wide range of study types, and there are subsections for both human subjects research and non-human subjects research. If any of the questions are not relevant to your research please mark them as "N/A" as appropriate.

Ethical considerations, permits and authorship

This section is applicable to all research types.

Provide details as to who granted permissions and/or consent for the study to take place in the Methods section of your manuscript. This should include the names of **all** ethics boards, governmental organizations, community leaders or other bodies that provided approval for the study. If individuals provided approval refer to these people by their role or title but do not list their name(s).

Reported on page number: 20

If there were any deviations from the study protocol after approval was obtained please provide details of these changes in the Methods section of your manuscript.

Reported on page number: N/A

Did this study involve local collaborators that are residents of the country where the research was conducted or members of the community studied? If you do not have any authors from said communities, please provide an explanation for this below.

This study did not involve local collaborators who are residents of the country where the research was conducted or members of the specific communities studied. The research primarily involved interviews with Universal Credit (UC) claimants and frontline staff working at local authorities, advice centres, housing associations, Jobcentre Plus, and a food bank in South London. The study was designed to capture administrative and claimant perspectives through a qualitative case study approach.

Everyone listed as an author should meet PLOS' criteria for authorship and all individuals who meet these criteria should be included in the author byline, rather than the acknowledgements. For further information please see the journal's Authorship Policy.

Human subjects research (e.g. health research, medical research, cross-cultural psychology)

Did you obtain written informed consent from a representative of the local community or region before the research took place? How did you establish who speaks for the community? Details of written informed consent obtained from study participants should be reported separately in the Methods section of your manuscript.

Yes, written informed consent was obtained from all participants before the study began. Participants were provided with detailed information regarding the study's objectives, procedures, potential risks, and their right to withdraw from the study at any time without penalty. Consent forms were signed by the participants, ensuring they understood the nature of the study and their rights. The research received ethical approval from the University of Nottingham Research Ethics Sub-Committee under the reference number 127-16-17-PGR.

However, the study did not involve obtaining consent from a representative of the local community or region. The study focused on individual-level experiences of Universal Credit claimants, and participants were recruited through a combination of purposive, snowball, and convenience sampling methods. Recruitment was facilitated by stakeholders in advice centres, Jobcentre Plus (JCP), and community organisations. The study did not rely on a single representative or community leader for consent because the objective was to capture diverse individual experiences rather than a unified community perspective.

Therefore, the informed consent process was conducted directly with individual participants rather than through a community representative. All participants had the right to ask questions about the study, seek clarification, and withdraw at any point without repercussions.

How did members of the local community provide input on the aims of the research investigation, its methodology, and its anticipated outcome(s)?

Members of the local community did not provide direct input on the aims of the research investigation, its methodology, or its anticipated outcomes. The research was designed based on an extensive review of existing literature on welfare conditionality and Universal Credit (UC), as well as policy frameworks and guidance from the Department for Work and Pensions (DWP). The study's aims and methodological approach were developed to explore the behavioural impacts of UC on claimants and frontline staff, focusing on benefit- and employment-related behaviours.

However, input from the local community was indirectly incorporated through the recruitment and interview process. Specifically:

- The study involved 32 UC claimants and 18 stakeholders (including frontline staff from local authorities, Jobcentre Plus, housing associations, and advice centres) in South London.
- These participants shared their personal experiences and perspectives on the implementation of UC, the challenges they faced, and the perceived effects on their behaviour.
- The methodology was refined through a pilot study, which involved interviews with a small number of participants to ensure the questions were clear and relevant. Feedback from these pilot interviews helped improve the data collection process.

Although there was no formal consultation with community representatives during the study design phase, the empirical data collected from individual participants directly shaped the analysis and interpretation of the study's findings. This ensured that the voices and experiences of the local community were accurately reflected in the research outcomes.

When engaging with the local community, how did you ensure that the informed consent documents and other materials could be understood by local stakeholders?

When engaging with the local community, ensuring that informed consent documents and other materials were accessible and understandable to stakeholders was a key priority. To achieve this, several steps were taken:

1. **Clear and Simple Language**: The informed consent documents, as well as other research materials, were written in clear and simple language to ensure they were easily understood by participants from diverse backgrounds. Technical or academic jargon was avoided, and explanations were provided in everyday language, making the content as straightforward as possible.
2. **Participant Information Sheets**: Detailed participant information sheets were provided alongside the consent forms. These sheets explained the purpose of the research, what participation involved, potential risks, the voluntary nature of participation, and the right to withdraw at any time without any consequence. The materials were carefully structured to facilitate easy comprehension.
3. **Assistance in Comprehension**: Before participants signed the informed consent forms, they were given the opportunity to ask questions about the study and the consent documents. If needed, the researcher or designated research assistants explained the documents in more detail to ensure participants understood their rights and the study's aims. This process was particularly important for participants who may have had limited literacy or language proficiency.
4. **Feedback and Clarification**: During the informed consent process, participants were encouraged to ask for clarification on any aspect of the study. This feedback was used to adjust materials where necessary to ensure that future participants also understood the content clearly.

These steps ensured that local stakeholders were able to provide fully informed consent, based on a clear understanding of the research aims, methodology, and potential outcomes. I have reflected this process in the revised Methods section of the manuscript.

Will the findings of the research be made available in an understandable format to stakeholders in the community where the study was conducted (e.g. via a presentation, summary report, copies of publications, etc.)? Please provide details of how this will be achieved.

Yes, the findings of the research will be made available to stakeholders in the community where the study was conducted, in an accessible and understandable format. The aim is to ensure that the results of the study are shared in a way that is meaningful and relevant to the local community. This will be achieved through the following strategies:

1. **Summary Report**: A concise summary report of the research findings will be prepared, highlighting the key outcomes and their implications for local stakeholders. This report will be written in clear, non-technical language, making it accessible to participants and other community members who may not have a background in social policy or research.
2. **Presentation to Stakeholders**: A presentation of the key findings will be delivered to relevant local stakeholders, including participants, community leaders, and organizations such as advice centres, Jobcentre Plus, and housing associations. This will provide an opportunity for stakeholders to ask questions, discuss the findings, and offer feedback. The presentation will be tailored to the audience, ensuring that it is engaging and relevant to their interests and concerns.
3. **Publications**: If appropriate, copies of any academic publications resulting from the research will be made available to stakeholders. These publications will be accompanied by an accessible summary of the findings, ensuring that the main results and their implications are easy to understand.
4. **Online Accessibility**: The summary report and other materials will be made available online, where stakeholders can easily access them. The research outcomes will be shared on websites of local community organizations, and links to the materials will be circulated via email or social media, ensuring wide distribution within the community.

By making the findings available in these various formats, we aim to ensure that the research is not only accessible but also useful and actionable for local stakeholders. This will help bridge the gap between academic research and community practice, allowing the findings to inform policy and improve local services.

Non-human subjects research using specimens/ animals collected as part of the study, or those housed in archival collections. Examples include archaeology, paleontology, botany and zoology.

Did the permission you obtained from a local authority to perform the study include an agreement on access to outputs and benefit sharing? This may include procedures to enable fair distribution of the benefits and resources arising from the research performed. Please include any details of Prior Informed Consent and Benefit Sharing Agreements obtained. These may be required by field-specific regulations, for example the Convention on Biological Diversity (CBD) and the associated Nagoya Protocol.

N/A

If the material used in your study was imported, please A) provide the year it was imported and B) indicate whether permits were obtained to import/export the materials used, C) provide details of any permits obtained. If this information is not available, please indicate this.

N/A

If you used archival specimens, please state how the material used in your study was acquired by the institute it is held in and provide details of any permits obtained for the original excavations/ sample collection. If this information is not available, please indicate this.

N/A

How was the potential cultural significance of the materials collected in your study to local communities considered in your research design? Were Indigenous peoples and/or local researchers and institutions involved with archaeological excavations / collection of specimens? If so, please provide a description of their involvement.

N/A

If your manuscript includes photographs of human remains please indicate whether authors obtained permission from descendants or affiliated cultural communities to do so.

N/A
